# When the messenger is more important than the message: an experimental study of evidence use in francophone Africa

**DOI:** 10.1186/s12961-022-00854-x

**Published:** 2022-05-26

**Authors:** Amandine Fillol, Esther McSween-Cadieux, Bruno Ventelou, Marie-Pier Larose, Ulrich Boris Nguemdjo Kanguem, Kadidiatou Kadio, Christian Dagenais, Valéry Ridde

**Affiliations:** 1 Department of Social and Preventive Medicine, School of Public Health (ESPUM), University of Montreal, Montreal, Canada; 2 CEPED, Université Paris cité - French National Research Institute for Sustainable Development (IRD), Paris, France; 3 Department of School and Social Adaptation Studies, Faculty of Education, Université de Sherbrooke, Sherbrooke, Canada; 4 French National Center for Scientific Research (CNRS), Aix-Marseille School of Economics, Aix Marseille University, Marseille, France; 5 INVEST Flagship Research Center/Department of Psychology and Speech-Language Pathology, University of Turku, Turku, Finland; 6 URMITE, French National Center for Scientific Research (CNRS), Aix-Marseille School of Economics - French National Research Institute for Sustainable Development (IRD), Dakar-Hann, Senegal; 7 Institute for Research in Health Sciences (IRSS), National Center for Scientific and Technologic Research, Ouagadougou, Burkina Faso; 8 RESILIENCE, French National Research Institute for Sustainable Development (IRD), Paris, France; 9 Department of Psychology, University of Montreal, Montreal, Canada; 10 Institut de santé et développement, Université Cheikh Anta Diop, Dakar, Senegal

**Keywords:** Global health, Policy briefs, Structural drivers, Power, Santé mondiale, Notes de politiques, Déterminants structurels, COVID-19, Pouvoir

## Abstract

**Background:**

Epistemic injustices are increasingly decried in global health. This study aims to investigate whether the source of knowledge influences the perception of that knowledge and the willingness to use it in francophone African health policy-making context.

**Methods:**

The study followed a randomized experimental design in which participants were randomly assigned to one of seven policy briefs that were designed with the same scientific content but with different organizations presented as authors. Each organization was representative of financial, scientific or moral authority. For each type of authority, two organizations were proposed: one North American or European, and the other African.

**Results:**

The initial models showed that there was no significant association between the type of authority or the location of the authoring organization and the two outcomes (perceived quality and reported instrumental use). Stratified analyses highlighted that policy briefs signed by the African donor organization (financial authority) were perceived to be of higher quality than policy briefs signed by the North American/European donor organization. For both perceived quality and reported instrumental use, these analyses found that policy briefs signed by the African university (scientific authority) were associated with lower scores than policy briefs signed by the North American/European university.

**Conclusions:**

The results confirm the significant influence of sources on perceived global health knowledge and the intersectionality of sources of influence. This analysis allows us to learn more about organizations in global health leadership, and to reflect on the implications for knowledge translation practices.

**Supplementary Information:**

The online version contains supplementary material available at 10.1186/s12961-022-00854-x.

## Résumé

**Contexte:**

Les injustices épistémiques sont de plus en plus décriées dans le domaine de la santé mondiale. Cette étude vise à déterminer si la source des connaissances influence la perception de ces connaissances et la volonté de les utiliser.

**Méthodes:**

L’étude suit un devis expérimental randomisé dans lequel les participant·es ont été assigné·es au hasard à l'une des sept notes politiques conçues avec le même contenu scientifique, mais avec différentes organisations présentées comme autrices. Chaque organisation était représentative d'une autorité financière, scientifique ou morale. Pour chaque type d'autorité, deux organisations étaient proposées : l'une nord-américaine ou européenne, l'autre africaine.

**Résultats:**

Les résultats montrent que le type d’autorité et la localisation des organisations autrices ne sont pas significativement associés à la qualité perçue et à l’utilisation instrumentale déclarée. Toutefois, des interactions entre le type d’autorité et la localisation étaient significatives. Ainsi, les analyses stratifiées ont mis en évidence que pour la qualité perçue, les notes de politique signées par l'organisme bailleur (autorité financière) africain obtenaient de meilleurs scores que les notes de politique signées par l’organisme bailleur nord-américain / européen. Tant pour la qualité perçue que pour l'utilisation instrumentale déclarée, ces analyses stratifiées ont révélé que les notes de politique signées par l'université africaine (autorité scientifique) étaient associées à des scores plus faibles que les notes de politique signées par l'université nord-américaine/européenne.

**Interprétation:**

Les résultats confirment l'influence significative des sources sur la perception des connaissances en santé mondiale et rappellent l’intersectionnalité de l’influence des sources d’autorité. Cette analyse nous permet à la fois d'en apprendre davantage sur les organisations qui dominent la scène de la gouvernance mondiale en santé et de réfléchir aux implications pour les pratiques d'application des connaissances.

## Background

Epistemic power issues are increasingly decried in global health because of their impacts on health inequalities [1–5]. Indeed, legitimation of knowledge is driven by economically dominant individuals or organizations [5–8] and elites guiding global decisions [9]. The lack of social diversity in the scientific, political or public health bodies is already unfair [10], but in addition, it contributes to the development of an *entre-soi* which excludes knowledge based on rationalities other than those commonly accepted. Going beyond traditional issues of social, material and geographical inequalities to explain health inequalities, Bhakuni and Abimbola [11] recently highlighted epistemic injustices in global health. Epistemic injustice was first defined by Fricker [12] as a wrong done to someone in his or her capacity as a knower. She described two types of epistemic injustice: testimonial and hermeneutical. First, testimonial injustice consists of discrediting the knowledge provided by someone due to the listener’s prejudices about his or her social characteristics. For example, during the Ebola epidemic in 2014, Lauer [13] highlighted how African experts were discredited by outside speakers and international actors. During the COVID-19 pandemic, Dalglish [14] decried “the lies given to global health expertise”, as there was a lack of recognition of African and Asian countries’ proactive measures in handling the crisis. Hermeneutical injustice is the impossibility that someone’s interpretation of a social phenomenon is recognized due to the lack of recognition of his or her world view by dominant groups. Bhakuni and Abimbola [11] called it interpretative injustice. For example, Lauer [13] explained that during the 2014 Ebola crisis, in North America, a strike in Sierra Leone was interpreted and mediated as the consequence of the selfish behaviour of health professionals rather than a demonstration against some health centre privatization by British expatriates. This mediatization contributed to devaluing the credibility of health professionals who participated in legitimate international interventions. While these two kinds of injustices are strongly related, the analysis we offer in this article aims to study testimonial injustice in global health. To our knowledge, no study has been conducted to identify testimonial injustices existing in global health. These injustices are often not seen, but they form the backdrop for global health governance and practices. They reveal discrimination against people discredited because of their social identity instead of their knowledge. This contributes to the generalization of the usual (non-epistemic) social discriminations.

A study carried out in Spain highlighted that credibility attributed to a scientific document differed depending on the organizations indicated as the authors [15]. In another study conducted on the YouTube platform, the perceived integrity and benevolence of the presenter of a science video had a significant influence on the perceived credibility of the information presented [16]. Furthermore, in another study, if the author was considered an authority in the relevant industry, then the reader was more likely to act on what was said in the document [17]. In this case, the presence of the author’s opinion also promoted a willingness to act or share information [17]. While scientists are supposed to be more experienced in dealing with issues of credibility of knowledge, it has been shown that they place more importance on “who is talking” than on “what is being said” [18].‬‬‬‬‬‬‬‬‬‬ In global health, there is growing confusion between scientific, expert, moral and financial authorities. For example, institutions with financial authority manage to legitimize knowledge and thus acquire an authority of expertise [19, 20]. At the same time, international organizations that are supposed to represent moral authorities are losing their credibility. WHO, for example, has been criticized for the influence of major donors in its decisions and for the confusion between different functions, notably between technical and political mandates [21, 22]. In addition, the lack of social diversity in international decision-making bodies as well as in academia, with an overrepresentation of men and individuals from North America or Europe, contributes to some voices not being heard [23]. This lack of representativeness leads to interpretative and testimonial epistemic injustices [24–27]. Testimonial injustices, which often occur toward minorities, are the product of those from low-income countries as much as those from high-income countries [28].

Francophone Africa is particularly representative of global health complexity, as the institutional landscape is composed of several actors from different sectors and countries involved in health policy. There are few studies dealing with epistemic injustices in this region, even though knowledge production and utilization are strongly influenced by power issues and colonization history [1, 11, 13, 27, 29, 30]. To our knowledge, there are no quantitative studies in this field that allow us to observe the effects of the source of a document on the perception of the quality of knowledge or with the intention to use it. As global health brings together actors from different sectors, countries and disciplines to offer solutions to health problems in the context of globalization, knowledge dissemination and translation are key mandates of global health stakeholders. Thus, the purpose of this research is to investigate whether the source of a document influences the perceived quality and the willingness to use knowledge contained in the document for global health stakeholders in francophone Africa.

### Present study

To conduct this research, we used a specific tool: the policy brief. Policy briefs are increasingly used to disseminate scientific findings to inform policy-makers of the best available knowledge [31]. A policy brief is “a concise document that prioritizes a particular policy issue and presents the evidence in nontechnical language and without professional jargon” [17]. The existing guides to support their design are quite varied, and few studies exist on their effectiveness [32].

Our main outcomes are perceived quality and reported instrumental use. First, credibility, while widely studied, is not the only quality attributed to knowledge that would improve its use. The visual aspect, the relevance of information and the recommendations in relation to local realities have been described as favourable to the effectiveness of policy briefs [32]. Perceived legitimacy, relevance and understanding are also qualities to be considered to improve the links between science, action and policy [33–35].

Use of knowledge is usually developed into three subcategories: (1) symbolic use, which aims to rely on, quote or argue from knowledge to legitimize a choice or a decision; (2) conceptual use, which is an increase in knowledge about a subject or issue and evolution in the understanding of it; and (3) instrumental use, which is associated with a change in practice or opinion [36, 37]. We chose to focus on instrumental use, as it is a concrete application of knowledge use for policy-making [38].

Therefore, this research aims to investigate whether the source of a policy brief influences not only its perceived credibility but also its perceived quality (visual appearance, relevance, legitimacy and comprehensibility) and reported instrumental use of knowledge for global health stakeholders in francophone Africa.

### Conceptual framework

To categorize the different sources (i.e. authoring organization), we borrow the typology of diffusion entrepreneur authorities [39]: financial, scientific, expertise and/or moral authority. To study the effects of the sources of a policy brief, we chose to use organizations representing different authorities as authoring organizations: (1) donor organizations for financial authority, (2) universities for scientific authority and (3) international organizations for moral authority. A typical organization representing expertise authority is more difficult to find, which is why we have limited ourselves to these three authorities. We also differentiated the authorities according to their geographical location (North America or Europe versus Africa) because many studies attest to the scientific hegemony of North America or Europe [13, 26, 30, 40, 41], especially in francophone Africa where these countries have a strong presence in the health landscape.

### Objectives and hypotheses

Our main objectives were to study two characteristics of the source of a policy brief: the type of authority and the location of the authority. We analysed (1) whether the type of authority (financial, scientific, moral) of the authoring organization of a policy brief is associated with perceived quality and reported instrumental use, (2) whether the location (North America or Europe versus Africa) of the authoring organization of a policy brief is associated with perceived quality and reported instrumental use, and (3) how the location of these authorities interacts with the type of authority in the prediction of perceived quality and reported instrumental use.

We did not have any hypothesis in regard to the influence of the types of authorities and the interaction between types of authorities and location. These analyses were exploratory given the lack of previous research on the topic among global health francophone African stakeholders.

However, we hypothesized that location would be a significant predictor, where authorities from North American or European countries would be associated with higher levels of perceived quality and reported instrumental use. We focused on these two regions (North America or Europe) because technical and financial partners involved in health decisions in francophone Africa are heavily represented by individuals from these regions. As exists other francophone areas in the world, we chose to focus on North America and Europe because of the financial and material power these countries exert in health policy-making and the influence such power can have on epistemic issues [13, 30, 40, 42, 43].

## Methods

### Study design

The study followed a randomized experimental design in which participants were randomly assigned to one of seven policy briefs that were designed with the same scientific content and visual features; only the authoring organizations were different. One of them contained no authoring organization.

The study was conducted remotely in three stages using a website specifically created for the study purpose (Wix website). A computer version and a mobile version were produced. First, the participant completed a first questionnaire. Second, the participant was randomly assigned to a policy brief that he or she had to read. The random distribution of the document was achieved using a programming code in HTML language in a window of the site (the code is available on request). Third, after reading the document, the participant completed a second questionnaire.

### Participants

#### Study population and sampling process

To be as representative as possible, we used the nine categories of global health actors proposed by Hoffman and Cole [44] to target the study participants: (1) national governments, (2) United Nations entities and international organizations, (3) development banks, (4) public/private partnerships, (5) philanthropic organizations, (6) global civil society and nongovernmental organizations, (7) private industries, (8) professional associations and (9) academic institutions. The countries involved in our study were French-speaking African countries (Benin, Burkina Faso, Chad, Guinea, Mali, Mauritania, Niger, Senegal and Togo) and the main countries involved in development assistance in this region (Belgium, Canada, France and Switzerland). Participants were contacted through our professional networks and through focal points in the countries or organizations. A table was constructed with the nine categories of actors for each country. For each case, one person (colleague, friend) was contacted and asked to transmit the study. Mailing lists and thematic groups on the links between science, policy and health were also used. As people are difficult to reach online, we relied on the snowball effect. People from all professional sectors (healthcare, policy, research, coordination, management, teaching, etc.) working in or with countries in francophone Africa were invited to participate. We expected a sample size between 200 and 300 participants which would allow a sufficient number of participants in the seven different policy brief conditions. Data collection was conducted from January to March 2021. We conducted exploratory analyses to determine whether we had enough participants and chose to stop data collection because of preliminary results that allowed for relevant analyses.

### Intervention

The intervention was the assignment of a policy brief where only the name and logo of the main author vary. To make the study as close to reality as possible, we used an existing policy brief (Additional files 1 and 2). This policy brief presented the results of a scoping review and recommendations related to the effectiveness of containment measures for vector-borne diseases and other emerging and re-emerging infectious diseases. Participants did not know what intervention they were assigned to.

### Measures

#### Main outcomes

The two main outcomes are the perceived quality of knowledge and its reported instrumental use. Questionnaires are available in Additional file 3.

##### Perceived quality of knowledge

The perceived quality of knowledge was measured through a set of statements (*n* = 10) for which participants expressed their level of agreement (Table 1). We identified complementary ways of considering the perceived quality of knowledge in the literature: visual appearance, credibility, legitimacy, relevance and comprehension. We therefore defined several statements to measure each of these constructs, thanks to studies already conducted on credibility [16, 45–48], legitimacy [49] and the visual aspect, relevance and understanding of the brief [17, 50–52]. For example, the following statement was offered to participants: “The content of the document is relevant to my work”, and participants responded on a five-point Likert scale: “Strongly disagree”, “Disagree”, “Agree”, “Somewhat agree”, “Strongly agree” or “Do not know or do not want to answer”. This last option was considered missing data in further analyses. We then assessed whether the set of statements allowed us to calculate an overall quality score. After examining the inter-item correlations and the distribution of each item, we calculated an overall perceived knowledge quality score by averaging the 10 statements (from 1 to 5). Cronbach’s alpha, which measures the internal consistency of the constructs in measuring the perceived quality score, was 0.88.

**Table 1:** Summary of variables to be measured and statements used

Outcomes	Dimensions	Statements
Perceived quality	Relevance	“The content of the document is relevant to my work.”
	Legitimacy	“The content of the paper is consistent with my professional value.” “The content of the paper seems to take into account a range of views and not just the author’s.”
	Credibility	“The level of detail provided in the document is appropriate.” “The methodology presented in the document appears robust.” “The rationale presented in the document leading to the recommendations is convincing.”
	Visual aspect	“The visual presentation of the document is attractive.” “The length of the document is adequate.”
	Comprehension	“The content of the document is easy to understand.” “The proposed recommendations are clear.”
Reported instrumental use		“Change my opinion on the issue of the effectiveness of measures to contain infectious disease outbreaks.” “Change my current policies or practices regarding the topic of containment measures to contain infectious disease outbreaks.” “Develop or sponsor new studies on the topic of containment measures to contain infectious disease outbreaks.”

##### Reported instrumental use of knowledge

Self-reported instrumental knowledge use was measured through a set of statements (*n* = 3) for which participants estimated the probability that they would perform different actions (Table 1). For example, they were asked, “Change my opinion on the effectiveness of measures to control infectious disease outbreaks”, and they responded on a five-point Likert scale: “Not at all likely”, “Unlikely”, “Likely”, “Somewhat likely”, “Very likely”, and “Do not know or do not want to answer”. This last option was considered missing data in further analyses. We proposed three items on changes in opinion, policy and practice. We then assessed whether the set of statements allowed us to calculate a score of reported instrumental use. After examining inter-item correlations and the distribution of each item, we calculated a score of reported instrumental knowledge use by averaging three statements (from 1 to 5; Cronbach’s alpha was equal to 0.78).

### Confounding variables

The following were considered confounding variables: prior knowledge about the topic of the policy brief before and after reading the policy brief (six questions), knowledge and opinion about the organization presented as the policy brief author (six questions), and sociodemographic, professional, geographical and migratory characteristics of the participants (10 questions).

### Analyses

#### Descriptive analyses

Descriptive analyses were conducted to observe the diversity of the sample, the distribution of conditions among participants and the main outcomes (perceived quality and reported instrumental use).

#### Main analyses

##### Are the type of authorities (financial, scientific and moral) and the location (North America or Europe versus Africa) of the authoring organization of a policy brief associated with perceived quality and reported instrumental use?

We conducted two linear regression models to observe whether the type of authorities (financial, scientific and moral) and location of the authoring organization were associated with our outcomes (i.e. perceived quality and reported instrumental use). We estimated our outcomes separately in two different models, but type of authorities and location of the authoring organization were entered in the same model, as we aimed to study the unique contribution of each factor. Professional experience (and perceived autonomy in the profession for the outcome reported instrumental use), gender, occupational sector, level of the last degree obtained and region where the last degree was obtained were systematically included in the models as covariates even when they were not significant, as they are among the socio-professional factors that are strongly related to the research question [17]. Knowing or not knowing the authoring organization was also included as a covariate. We believe that knowledge of the organization is a potential intervening variable that can explain the relationships between the authoring organization and the different dependent variables.

##### How the type of authority (financial, scientific and moral) of authoring organization interacts with the location of these authorities (North America or Europe versus Africa) in the prediction of perceived quality and reported instrumental use?

First, we added an interaction term to our previous linear regression models between the variables of type of authority and the location of these authorities. We tested separately, in two different models, these interactions in relation to perceived quality and reported instrumental use. When we detected a significant interaction at the 10% significance level, we carried out stratified analyses where we regressed the location of the authorities on the perceived quality and reported instrumental use according to the type of authority of the authoring organizations (financial, scientific, moral).

## Results

### Descriptive analyses

#### Participant characteristics

The sample consisted of 233 participants, the majority of whom were aged between 26 and 45 years (64%, *n* = 148/233) and were male (68%, *n* = 159/233). The majority had a graduate degree (88%, *n* = 205/233). The professions represented were mainly project leaders, managers or coordinators (30%, *n* = 70/233), researchers (24%, *n* = 57/233) and health professionals (18%, *n* = 43/233). Their experience ranged from 0 to over 25 years. Regarding geographical distribution, the main birthplaces were France (23%, *n* = 54/233), Mali (15%, *n* = 35/233), Burkina Faso (11%, *n* = 25/233) and Benin (10%, *n* = 22/233). For education, the main places where the highest degree was obtained were France (35%, *n* = 82/233) or Mali (10%, *n* = 24/233). More than one third of the participants were born, educated and lived in West or Central Africa (36%, *n* = 88/233), and less than a fifth were born, educated and lived in Europe or North America (16%, *n* = 39/233). A small percentage of participants (13%, *n* = 31/233) were born and resided in West or Central Africa and obtained their highest degree in Europe or North America. Finally, less than one tenth (8%, *n* = 20/233) were born and graduated with their highest degree in Europe or North America and lived in West or Central Africa at the time of the survey. A detailed description of the participants is presented in Additional file 4.

#### Distribution of conditions among participants and outcome description

Policy briefs signed by the African donor organization were the least frequently assigned to the participants (*n* = 24) and policy briefs not signed by any author were the most frequently assigned to the participants (*n* = 39) (Table 2).

**Table 2:** Intervention distribution and outcome description

	Perceived quality score			Reported instrumental use score		
	n	Mean (/5)	Standard deviation	n	Mean (/5)	Standard deviation
Authoring organization						
Donor organizations	63	4.005	0.547	64	3.339	1.086
North American/European donor organization	30	3.943	0.460	31	3.226	1.023
African donor organization	33	4.062	0.617	33	3.444	1.148
International organization	56	3.944	0.803	57	3.705	0.897
North American/European office of international organization	33	4.073	0.813	33	3.849	0.791
African office of international organization	23	3.759	0.768	24	3.507	1.009
Universities	72	3.926	0.623	72	3.301	1.060
North American/European university	38	4.196	0.554	38	3.640	0.973
African university	34	3.625	0.562	34	2.922	1.038
No author	37	3.657	0.791	39	3.111	0.938
Total	228	3.909	0.687	232	3.379	1.024

Participants rated the policy brief as adequate in terms of quality (mean: 3.909/5, standard deviation: 0.687). In terms of reported instrumental use, participants mainly reported that they would be likely to use the policy brief (mean: 3.379/5, standard deviation: 1.024) (Table 2).

For perceived quality, the lowest score was assigned to a policy brief signed by the African university (mean: 3.625/5, standard deviation: 0.562), and the highest score was assigned to a policy brief signed by the North American/European university (mean: 4196/5, standard deviation: 0.554). For reported instrumental use, the lowest score was assigned to a policy brief signed by the African university (mean: 2.922/5, standard deviation: 1.038), and the highest score was assigned to a policy brief signed by the North American/European office of the international organization (mean: 3.849/5, standard deviation: 0.791) (Table 2).

#### Main analyses

##### Are the type of authority and the location of the authoring organization of a policy brief associated with the perceived quality of knowledge?

An initial model found that perceived quality was not significantly associated with the type of authority or the location of the authoring organization. Knowledge of the organization was not significantly associated with the perceived quality score.

The perceived quality score was significantly increased by 0.423 when participants graduated from a North America/European institution compared to those who graduated from an African institution (β = 0.423, 95% CI = 0.082 to 0.764, *P* = 0.015) (Table 3).

**Table 3:** Associations between type of authority and location of the authoring organization, and perceived quality of knowledge

	β	Perceived quality		*P* value
		[95% CI]		
Intervention				
Type of authority of authoring organization				
Donor organization (financial authority)	Ref.	Ref.		Ref.
International organization (moral authority)	−0.227	[−0.612; 0.158]		0.248
University (scientific authority)	−0.073	[−0.448; 0.303]		0.704
Location of authoring organization				
Europe or North America	Ref.	Ref.		Ref.
Africa	−0.508	[−0.246; 0.348]		0.738
Confounding variable				
Prior knowledge of the organization				
Does not know the organization	Ref.	Ref.		Ref.
Knows the organization	0.213	[−0.179; 0.605]		0.287
Experience in the profession	−0.092	[−0.238; 0.055]		0.221
Gender				
Male	Ref.	Ref.		Ref.
Female	−0.120	[−0.443; 0.203]		0.468
Profession				
Health professionals	Ref.	Ref.		Ref.
Other	−0.165	[−0.695; 0.366]		0.542
Coordination/management of programmes	−0.262	[−0.742; 0.217]		0.284
Research professionals	−0.328	[−0.822; 0.167]		0.194
Level of study				
University level less than or equal to first cycle	Ref.	Ref.		Ref.
University level second cycle (MD)	0.073	−0.390	0.536	0.757
University level postgraduate (PhD)	−0.339	−0.809	0.130	0.157
Region of graduation				
Europe or North America	Ref.	Ref.		Ref.
Africa	0.423	[0.082; 0.764]		0.015*
	Value			
*R*^2^	0.155			
Adjusted *R*^2^	0.077			

##### Are the type of authority and the location of the authoring organization of a policy brief associated with reported instrumental use?

An initial model found that perceived quality was not significantly associated with the type of authority or the location of the authoring organization. Knowledge of the organization was not significantly associated with the perceived quality score.

The reported instrumental use score was significantly lower by 0.496 when participants were coordinators/managers of health programmes and lower by 0.542 when they were research professionals compared to health professionals (respectively β = −0.496, 95% CI = −0.992 to −0.001, *P* = 0.050 and β = −0.542, 95% CI = −1.057 to −0.281, *P* = 0.039). It was also significantly lower by 0.542 when participants had a postgraduate level of education compared to participants with a university level less than or equal to the first cycle (β = 0.542, 95% CI = −1.025 to −0.584, *P* = 0.028). Participants who graduated from an African university reported a higher score of instrumental use by 0.739 compared to those who graduated from a European or North American university (β = 0.739, 95% CI = 0.839 to 1.089, *P* < 0.001) (Table 4).

**Table 4:** Associations between authoring organization and reported instrumental use of knowledge

	β	Reported instrumental use	*P* value
		[95% CI]	
Intervention			
Type of authority of authoring organization			
Donor organization (financial authority)	Ref.	Ref.	Ref.
International organization (moral authority)	0.161	[−0.234; 0.557]	0.420
University (scientific authority)	−0.128	[−0.514; 0.259]	0.514
Location of authoring organization			
Europe or North America	Ref.	Ref.	Ref.
Africa	−0.083	[−0.390; 0.224]	0.593
Confounding variable			
Prior knowledge of the organization			
Does not know the organization	Ref.	Ref.	Ref.
Knows the organization	0.145	[−0.268; 0.558]	0.489
Perceived autonomy in the profession	0.751	[−0.051; 0.202]	0.242
Experience in the profession	0.166	[−0.133; 0.166]	0.827
Gender			
Male	Ref.	Ref.	Ref.
Female	−0.322	[−0.650; 0.005]	0.053
Profession			
Health professionals	Ref.	Ref.	Ref.
Other	−0.463	[−1.009; 0.823]	0.095
Coordination/management of programmes	−0.496	[−0.992; −0.001]	0.050*
Research professionals	−0.542	[−1.057; −0.281]	0.039*
Level of study			
University level less than or equal to first cycle	Ref.	Ref.	Ref.
University level second cycle (MD)	−0.205	[−0.682; 0.271]	0.395
University level postgraduate (PhD)	−0.542	[−1.025; −0.584]	0.028*
Region of graduation			
Europe or North America	Ref.	Ref.	Ref.
Africa	0.739	[0.389; 1.089]	0.000*
	Value		
*R*^2^	0.323		
Adjusted *R*^2^	0.253		

##### How the type of authority of authoring organization interacts with the location of these authorities in the prediction of perceived quality?

We found that the interaction between international organizations and location was not significant (β = −0.004, 95% CI = −0.754 to 0.763, *P* = 0.991), but the interaction between universities and location was significant (β = −1.517, 95% CI = −2.225 to −0.810, *P* < 0.001). Therefore, we performed a stratified analysis by type of authority of the authoring organization.

###### Donor organization

The first stratified model revealed that the perceived quality score was increased by 0.510 when participants received policy briefs signed by the African donor organization compared to those who received policy briefs signed by the North American/European donor organization (β = 0.510, 95% CI = 0.061–0.960, *P* = 0.027) (Table 5). In other words, for a mean score of perceived quality for policy briefs signed by the North American/European donor organization equal to 3.943 (Table 2), the score of perceived quality was 12.9% higher for participants who received policy briefs signed by the African donor organization.

**Table 5:** Associations between location of authoring organization and perceived quality, stratified by sector of authoring organization

	β	Perceived quality	*P* value
		[95% CI]	
**Donor organizations (financial authority)**			
Intervention			
Location of authoring organization			
Europe or North America	Ref.	Ref.	Ref.
Africa	0.510	[0.061 to 0.960]	0.027*
Confounding variable			
Prior knowledge of the organization			
Does not know the organization	Ref.	Ref.	Ref.
Knows the organization	0.413	[−0.473 to 1.300]	0.350
Experience in the profession	0.084	[−0.180 to 0.348]	0.520
Gender			
Male	Ref.	Ref.	Ref.
Female	−0.171	[−0.658 to 0.317]	0.482
Profession			
Health professionals	Ref.	Ref.	Ref.
Other	−0.523	[−1.256 to 0.211]	0.157
Coordination/management of programmes	−0.209	[−0.871 to 0.454]	0.454
Research professionals	−0.591	[−1.441 to 0.260]	0.260
Level of study			
University level less than or equal to first cycle	Ref.	Ref.	Ref.
University level second cycle (MD)	0.505	[−0.231 to 1.241]	0.172
University level postgraduate (PhD)	0.582	[−0.148 to 1.312]	0.114
Region of graduation			
Europe or North America	Ref.	Ref.	Ref.
Africa	0.377	[−0.130 to 0.883]	0.140
	Value		
*R*^2^	0.337		
Adjusted *R*^2^	0.141		
**Regional offices of international organization (moral authority)**			
Intervention			
Location of authoring organization			
Europe or North America	Ref.	Ref.	Ref.
Africa	0.581	[−0.093 to 1.256]	0.089
Confounding variable			
Prior knowledge of the organization			
Does not know the organization	Ref.	Ref.	Ref.
Knows the organization	0.123	[−0.763 to 1.008]	0.780
Experience in the profession	−0.361	[−0.714 to −0.009]	0.045*
Gender			
Male	Ref.	Ref.	Ref.
Female	−0.655	[−1.287 to −0.024]	0.042*
Profession			
Health professionals	Ref.	Ref.	Ref.
Other	0.207	[−1.062 to 1.477]	0.742
Coordination/management of programmes	−0.590	[−1.612 to 0.442]	0.255
Research professionals	−0.694	[−1.718 to 0.330]	0.177
Level of study			
University level less than or equal to first cycle	Ref.	Ref.	Ref.
University level second cycle (MD)	0.247	[−0.641 to 1.135]	0.576
University level postgraduate (PhD)	−0.590	[−1.454 to 0.274]	0.174
Region of graduation			
Europe or North America	Ref.	Ref.	Ref.
Africa	0.655	[−0.982 to 1.409]	0.086
	Value		
*R*^2^	0.506		
Adjusted *R*^2^	0.361		
**Universities (scientific authority)**			
Intervention			
Location of authoring organization			
Europe or North America	Ref.	Ref.	Ref.
Africa	−0.886	[−1.361 to −0.412]	0001*
Confounding variable			
Prior knowledge of the organization			
Does not know the organization	Ref.	Ref.	Ref.
Knows the organization	0.210	[−0.280 to 0.700]	0.393
Experience in the profession	−0.124	[−0.334 to 0.086]	0.239
Gender			
Male	Ref.	Ref.	Ref.
Female	−0.056	[−0.629 to 0.516]	0.843
Profession			
Health professionals	Ref.	Ref.	Ref.
Other	−0.505	[−1.327 to 0.317]	0.222
Coordination/management of programmes	−0.289	[−1.045 to 0.467]	0.446
Research professionals	−0.287	[−1.101 to 0.438]	0.429
Level of study			
University level less than or equal to first cycle	Ref.	Ref.	Ref.
University level second cycle (MD)	−0.450	[−1.323 to 0.425]	0.305
University level postgraduate (PhD)	−0.910	[−1.811 to −0.010]	0.048*
Region of graduation			
Europe or North America	Ref.	Ref.	Ref.
Africa	−0.039	[−0.639 to 0.560]	0.896
	Value		
*R*^2^	0.375		
Adjusted *R*^2^	0.230		

###### International organization

There were no significant differences by location of authoring organization for policy briefs signed by international organization (Table 5).

###### University

The third stratified model revealed that perceived quality score was lower by 0.886 when participants received policy briefs signed by the African university compared to those who received policy briefs signed by the North American/European university (β = −0.886, 95% CI = −1.361 to −0.412, *P* = 0.001) (Table 4). In other words, for a mean score of perceived quality for policy briefs signed by the North American/European university equal to 4.196 (Table 2), the score of perceived quality was 21.1% lower for participants who received policy briefs signed by the African university.

##### How the type of authority of authoring organization interacts with the location of these authorities in the prediction of reported instrumental use?

We found that the interaction between international organizations and location was not significant (β = −0.057, 95% CI = −0.815 to 0.702, *P* = 0.883), but the interaction between universities and location was significant (β = −1.110, 95% CI = −1.822 to 0.398, *P* = 0.002). Therefore, we performed a stratified analysis by type of authority of the authoring organization.

###### Donor organization

There were no significant differences by location of authoring organization for policy briefs signed by donor organizations (Table 6).

**Table 6:** Associations between location of authoring organization and reported instrumental use of knowledge, stratified by sector of authoring organization

	β	Reported instrumental use	*P* value
		[95% CI]	
**Donor organizations (financial authority)**			
Intervention			
Location of authoring organization			
Europe or North America	Ref.	Ref.	Ref.
Africa	0.373	[−0.119 to 0.865]	0.132
Confounding variable			
Prior knowledge of the organization			
Does not know the organization	Ref.	Ref.	Ref.
Knows the organization	1.064	[−0.906 to 2.219]	0.070
Perceived autonomy in the profession	−0.010	[−0.203 to 0.182]	0.913
Experience in the profession	0.239	[0.040 to 0.617]	0.027
Gender			
Male	Ref.	Ref.	Ref.
Female	−0.582	[−1.100 to −0.641]	0.029*
Profession			
Health professionals	Ref.	Ref.	Ref.
Other	−0.793	[−1.632 to 0.464]	0.063
Coordination/management of programmes	−0.381	[−1.116 to 0.535]	0.298
Research professionals	−0.505	[−1.410 to 0.400]	0.264
Level of study			
University level less than or equal to first cycle	Ref.	Ref.	Ref.
University level second cycle (MD)	0.150	[−0.639 to 0.940]	0.701
University level postgraduate (PhD)	−0.279	[−1.052 to 0.493]	0.466
Region of graduation			
Europe or North America	Ref.	Ref.	Ref.
Africa	1.099	[0.574 to 1.624]	0.000*
	Value		
*R*^2^	0.644		
Adjusted *R*^2^	0.518		
**Regional offices of international organization (moral authority)**			
Intervention			
Location of authoring organization			
Europe or North America			
Africa	0.458	[−0.074 to 0.990]	0.089
Confounding variable			
Prior knowledge of the organization			
Does not know the organization			
Knows the organization	0.225	[−0.464 to 0.915]	0.510
Perceived autonomy in the profession	−0.010	[−0.227 to 0.208]	0.928
Experience in the profession	−0.368	[−0.638 to −0.098]	0.009*
Gender			
Male			
Female	−0.470	[−0.952 to 0.011]	0.055
Profession			
Health professionals			
Other	−0.248	[−1.214 to 0.718]	0.605
Coordination/management of programmes	−0.856	[−1.643 to −0.068]	0.034*
Research professionals	−0.962	[−1.762 to −1.162]	0.020*
Level of study			
University level less than or equal to first cycle			
University level second cycle (MD)	−0.357	[−1.075 to 0.296]	0.256
University level postgraduate (PhD)	−0.449	[−1.123 to 0.225]	0.184
Region of graduation			
Europe or North America			
Africa	0.357	[−0.230 to 0.944]	0.225
	Value		
*R*^2^	0.549		
Adjusted *R*^2^	0.394		
**Universities (scientific authority)**			
Intervention			
Location of authoring organization			
Europe or North America			
Africa	−0.670	[−1.265 to −0.748]	0.028*
Confounding variable			
Prior knowledge of the organization			
Does not know the organization			
Knows the organization	0.038	[−0.601 to 0.677]	0.906
Perceived autonomy in the profession	0.183	[−0.068 to 0.435]	0.148
Experience in the profession	−0.057	[−0.325 to 0.211]	0.669
Gender			
Male			
Female	−0.185	[−0.905 to 0.536]	0.608
Profession			
Health professionals			
Other	−0.325	[−1.360 to 0.710]	0.530
Coordination/management of programmes	−0.538	[−1.493 to 0.417]	0.262
Research professionals	−0.514	[−1.441 to 0.412]	0.269
Level of study			
University level less than or equal to first cycle			
University level second cycle (MD)	−0.299	[−1.403 to 0.804]	0.587
University level postgraduate (PhD)	−0.316	[−1.453 to 0.820]	0.577
Region of graduation			
Europe or North America			
Africa	0.530	[−0.270 to 1.330]	0.188
	Value		
*R*^2^	0.313		
Adjusted *R*^2^	0.129		

###### International organization

There were no significant differences by location of authoring organization for policy briefs signed by the international organization (Table 6).

###### University

The third stratified model revealed that reported instrumental use score was lower by 0.670 when participants received policy briefs signed by the African university compared to those who received the policy briefs signed by the North American/European university (β = −0.670, 95% CI = −1.265 to −0.748, *P* = 0.028) (Table 6). In other words, for a mean score of reported instrumental use for policy briefs signed by the North American/European university equal to 3.640 (Table 2), the score of perceived quality was 18.4% lower for participants who received policy briefs signed by the African university.

## Discussion

### Summary of results

Initial linear regression models showed that the type of authority (financial, scientific, moral) and the location (North America/Europe, Africa) of the policy brief authoring organization were not significantly associated with perceived quality and reported instrumental use.

Stratified analyses highlighted that policy briefs signed by the African donor organization were perceived to be of higher quality than policy briefs signed by the North American/European donor organization. These analyses also found that policy briefs signed by the African university were associated with lower scores for both perceived quality and reported instrumental use than policy briefs signed by the North American/European university.

With respect to social characteristics, results show that participants who graduated in Africa both perceived better quality and reported more instrumental use than participants who graduated in North America/Europe. For reported use, the results show that participants working as coordinators or managers of health programmes and research professionals reported less use than health professionals, and similarly postgraduate participants reported less than participants with university study level less than or equal to the first cycle.

### Reputation heuristics and unfair evaluation of knowledge

We believe that “reputation heuristics”, which have been studied in other contexts, can lead to unfair evaluation of online information [47], and consequently to testimonial injustice. We saw that the knowledge contained in policy briefs signed by the African donor organization was perceived to be of higher quality than those signed by the North American/European donor organization, and the opposite trend was observed for policy briefs signed by universities. This difference could have been explained by a “knowledge bias”, which would have made it possible to associate an organization that one knows with a better perception of its activities, but this was not the case.

#### Lack of confidence in the North American/European donor organization?

The involvement of foreign donor organizations in francophone African countries has long been strongly criticized. Indeed, on the one hand, the financial and political stakes raise questions about the value underlying the interventions of “external partners” in this historical area. On the other hand, the lack of knowledge of the terrain and the interventions they implement lead to a questioning of their legitimacy [53]. There is also a problem of capitalizing on acquired knowledge and experience to promote learning and improve practices [54]. There is distrust about the links between policy and knowledge production [55, 56], which was observed in particular in the processes of knowledge used for the fight against COVID-19 that are not specific to the context between France and West Africa. Specific distrust exists regarding the intervention of external agencies in this region, which could undermine the perception of quality or the willingness to use the policy notes signed by the northern donor organizations. Despite the increased visibility of northern donor organizations in the francophone African landscape, their presence is not associated with a better perception of the quality of knowledge production or the willingness to use it.

#### A vicious cycle between testimonial and interpretative injustices for francophone African universities?

Francophone African universities, suffering from a lack of public investment, may be victims of a decline in scientific authority [57]. The low recognition of francophone African universities in scientific activities is often due to (among other things) a lack of professors and researchers, a lack of doctoral training and a lack of social recognition of the research activity [58–60]. In the most widely used world university rankings (such as the *Shanghai Ranking* or the *Times Higher Education*), these universities are not even listed. When they are listed, they do not appear before the 2000th place. These rankings, rather than providing an idea of the levels of universities, exclude some from the institutional research landscape [61]. This is not a brutal and material exclusion, but a soft and diffuse one because of the development of a hierarchy of universities and knowledge. The problem is that even though the lack of public investment is a structural determinant of knowledge production quality, the generalization of the representation of the low quality of knowledge or research activity in francophone African universities can lead to a vicious cycle between interpretative, testimonial and social injustices. Indeed, because of their low capacity to mobilize financial resources and their weak international influence, francophone African universities have little influence on the agendas of global governance, and the “monetary” use of knowledge may reinforce the mutual dependence of epistemic and social injustices.

### A “monetary” use of knowledge?

While the policy briefs signed by the North American/European donor organization were perceived as being of lower quality, they were not reported as being less used than the policy briefs signed by the African donor organization. When considering the use of knowledge in the political sector, knowledge may be evaluated on the basis of the author's reputation [62] or the degree of agreement with the author [49] rather than the knowledge content. This may be explained by the idea that knowledge presented by organizations with financial authority is more useful in advancing an idea, putting an issue on the agenda or organizing resistance to a policy [63], particularly in the “development arena” [64]. Indeed, often referred to as “technical and financial partners”, donor and international organizations participate in national health committees in francophone African countries [65] in the same way as national organizations, which gives them power in the development of health policies. At the same time, universities, already suffering underinvestment from public policies, are being supplanted by consultancy activities in the production of knowledge [59, 66–68], which contributes to the decline in the monetary value of the knowledge produced by universities. The “deep core” of neoliberalism also plays out at this level. Indeed, it both guides the education sector in the same way that it guides the health sector, influencing the criteria for university rankings [61, 69], and participates in the commodification of knowledge to be increasingly “useful” [27]. Individuals may seek to use knowledge that they find more useful, in a monetary sense, particularly in the development or global health arena. While this “monetization” of knowledge use can strengthen the authority of dominant organizations, even if the quality of knowledge is perceived as poor, it is detrimental to organizations that do not have financial or moral power, and it can serve to reinforce the intersectionality of normative, financial and epistemic powers.

### Limits and implications for knowledge translation

It would be useful to complement this quantitative analysis with a qualitative study to compare the explanations given by stakeholders for these results. Indeed, this study is exploratory, and it is still difficult to propose rigorous explanations of the results. In addition, this study was offered online in a context where containment measures during the COVID-19 pandemic were being implemented in several countries. Currently, the flow of information via digital means has increased. Participants may not have taken the necessary care to complete the questionnaire thoroughly. The topic of containment effectiveness was chosen intentionally to increase the willingness of the individuals contacted to participate. The method of contact, through mailing lists and focal points, may have also had an impact on the sample, which is not as diverse as if participants had been contacted through national sampling frames or institutional directories. It is also possible that the fact that the first author of this analysis presented herself as a doctoral student and member of a northern research institute to increase participation in the survey may have influenced the perceived source of the policy briefs. It would have been very interesting to increase the number of scenarios with a larger number of organizations, but the number of participants would not have allowed for statistically sound analyses. A last limit is that we did not ensure that the participants had no previous knowledge of the policy brief, and if they recognized the name of the authoring organization, it may have had an influence on the results.

This analysis permits us to both learn more about organizations in global health leadership and reflect on the implications for knowledge translation practices. We have seen that participants with their highest degree from African universities are more likely to report using knowledge than those with their highest degree from institutions in Europe or North America, and similarly for men versus women or graduate versus postgraduate. This difference in attitude might be better understood through further qualitative analysis. This could allow us to try to adapt strategies that consider the rationalities of individuals according to their social characteristics, rather than strategies by context, without considering the differences between social groups [70]. In addition, a larger number of participants would have allowed us to examine interactions between regions of education, gender and authoring organizations to better understand how and why individuals use or do not use knowledge and the dynamics with organizational authority issues in the global health context.

## Conclusion

The results confirm the significant influence of sources on perceived global health knowledge. This study is exploratory, and further analysis would be useful to better understand the dynamics between authoring organization representation, perceived quality and knowledge use to take them into account in knowledge translation strategies.

## Supplementary Information

**Additional file 1:** Policy brief initial version (available on: https://36671ce8-37d7-4c16-9292-45ae340934dc.filesusr.com/ugd/a01b06_85eeaabf27084f02abe52517c705ef66.pdf).

**Additional file 2:** First and last page of modified policy brief.

**Additional file 3:** Survey.

**Additional file 4:** Description of the participants.

## Data Availability

The datasets generated and/or analysed during the current study are not publicly available due to the necessity of authoring organization anonymity but are available from the corresponding author on reasonable request.
