# Supplementary material for: When the messenger is more important than the message: an experimental study of evidence use in francophone Africa
Source: Health Res Policy Syst. 2022 May 26;20:57. doi: 10.1186/s12961-022-00854-x (PMC9134721; doi:10.1186/s12961-022-00854-x)
Supplement: Supplementary file 1 — Additional file 1: Policy brief initial version (available on: https://36671ce8-37d7-4c16-9292-45ae340934dc.filesusr.com/ugd/a01b06_85eeaabf27084f02abe52517c705ef66.pdf). [file 12961_2022_854_MOESM1_ESM.docx]

Additional file 1 : Policy brief initial version (available on : <https://36671ce8-37d7-4c16-9292-45ae340934dc.filesusr.com/ugd/a01b06_85eeaabf27084f02abe52517c705ef66.pdf>)
