# Supplementary material for: When the messenger is more important than the message: an experimental study of evidence use in francophone Africa
Source: Health Res Policy Syst. 2022 May 26;20:57. doi: 10.1186/s12961-022-00854-x (PMC9134721; doi:10.1186/s12961-022-00854-x)
Supplement: Supplementary file 2 — Additional file 2: First and last page of modified policy brief. [file 12961_2022_854_MOESM2_ESM.pdf]

File 2 : Modified policy brief

Logo de l'organisation autrice

LES MESURES DE CONFINEMENT FONCTIONNENT-ELLES POUR LES MALADIES À TRANSMISSION VECTORIELLE ET D’AUTRES MALADIES INFECTIEUSES ÉMERGENTES ET RÉ-ÉMERGENTES ?

UNE NOTE DE RECHERCHE À L’INTENTION DES DÉCIDEURS POLITIQUES MONDIAUX ET NATIONAUX

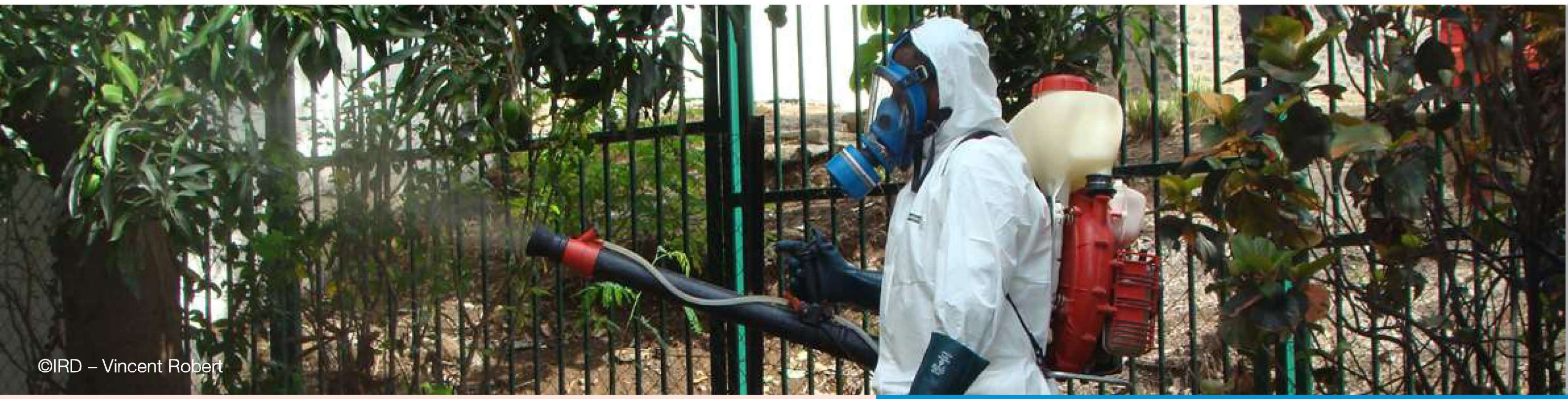
©IRD – Vincent Robert

RÉSUMÉ

Plusieurs mesures visant à endiguer les épidémies de maladies infectieuses sont en place à l’échelle mondiale. Toutefois, leur mise en œuvre et leur efficacité sont insuffisamment répertoriées. Nous avons réalisé un examen de la portée des mesures de confinement pour les maladies à transmission vectorielle (MTV) et d’autres maladies infectieuses émergentes et ré-émergentes. Les articles examinés (n=31) ont démontré que les stratégies de confinement étaient avant tout conçues et lancées une fois les épidémies déjà déclarées et établies. La majorité des études présentaient des expériences portant sur la prestation de services de soins et des interventions environnementales ou à caractère sanitaire, avec peu d’interventions à base communautaire. Elles ne comportaient aucun renseignement sur des pratiques standardisées, des processus de mise en œuvre ou des modifications apportées aux interventions initialement conçues. L’évaluation de l’efficacité se faisait généralement par observation et était rarement de nature expérimentale. Les recommandations présentées dans les publications servaient à créer une liste de recommandations à l’intention des parties prenantes qui pouvaient ensuite être utilisées pour concevoir ou mettre en œuvre ultérieurement des directives propres à la gestion des épidémies.

CE QU’IL FAUT RETENIR

- **En présence de maladies cycliques ou saisonnières, les stratégies de confinement des épidémies doivent être planifiées ou conçues au préalable, dans la mesure du possible.**
- **La consignation systématique du modèle et du mode de mise en œuvre des mesures de confinement est nécessaire pour améliorer la création de directives standardisées à usage général.**
- **Il a été rapporté que l’amélioration de la structure du système de santé, notamment la formation des prestataires de soins, de la surveillance, et de la gestion et de l’affectation des ressources (lits, médicaments, etc.) constituaient des mesures efficaces pour endiguer les épidémies.**
- **L’inclusion de mesures environnementales et sanitaires, telles que la décontamination, la mise en quarantaine et la brumisation ont été citées parmi les mesures de confinement les plus fréquemment utilisées.**

Description de l’organisation autrice

Logo de l’organisation autrice

RECOMMANDATIONS

1er Focalisez-vous sur les approches proactives, lorsque le temps et les ressources le permettent. Par exemple :

- → Reconnaissez et identifiez la présence d'un foyer de maladie au moment opportun.
- → Développez et préparez un plan de base générique pour lutter contre l'éclosion de la maladie en incluant la possibilité de procéder à une réaffectation des ressources économiques et humaines, le cas échéant.
- → Identifiez les états de santé cycliques et saisonniers (par ex. dengue ou paludisme après la saison des pluies) et produisez une procédure cadre évaluant le rôle de surveillance et la disponibilité des lits d'hôpitaux.

2e Favorisez la formation des professionnels de santé et l'amélioration des infrastructures médicales durant les périodes inter-épidémiques de menaces connues. Par exemple, la création de plans pour la formation du personnel médical, en charge de la surveillance et de l'assainissement sur les maladies connues et ré-émergentes contribue à l'amélioration du système de santé, comme cela a pu être observé dans les interventions les plus abouties.

3e Appuyez-vous sur des cadres d'action qui ont porté leurs fruits par le passé ou dans des contextes similaires (en d'autres termes, lorsque vous planifiez des interventions, fiez-vous à des recommandations étayées par des données probantes concernant des situations similaires à celles rencontrées dans votre contexte local).

4e Cherchez à promouvoir une description globale de votre intervention, en particulier s'agissant du contexte, en utilisant des listes de vérification validées.

- → Attribuez des ressources et du temps aux responsables du secteur de la santé publique pour qu'ils rapportent de manière globale et systématique le modèle et le mode de mise en œuvre des mesures de confinement.
- → Incluez une période d'évaluation adéquate dans la planification des interventions.

5e Cherchez à promouvoir une participation communautaire sur la durée, dans la mesure où cette démarche favorise son implication avant et après l'éclosion des maladies.

Logo de l'organisation autrice
