# Supplementary material for: When the messenger is more important than the message: an experimental study of evidence use in francophone Africa
Source: Health Res Policy Syst. 2022 May 26;20:57. doi: 10.1186/s12961-022-00854-x (PMC9134721; doi:10.1186/s12961-022-00854-x)
Supplement: Supplementary file 3 — Additional file 3: Survey. [file 12961_2022_854_MOESM3_ESM.pdf]

Additional file 3 : Survey

Question	Responses
**Participant presentation (before reading)**	
Socio-demographic characteristics	
1. Are you …	Male / Female / Other / I do not wish to answer
2. How old are you ?	Less than 25 years old / 26 to 35 years old / 36 to 45 years old / 46 to 55 years old / 56 to 65 years old / More than 65 years old / I do not wish to answer
3. What is your level of education? (Last degree obtained)	Baccalaureate or less / 1st cycle university level (Licence, DEUG) / 2nd cycle university level (Maîtrise, Master 1 or 2) / 3rd cycle university level (Doctorat) / Other / I do not wish to answer
Profession	
4. In which type of organization do you mainly work?	Academic institution or research center / Government or ministry / Government agencies (e.g. Institute of Public Health, etc.) / Health center (e.g. Hospitals, clinics, etc.) / Non-governmental organization / Civil society organization (local or international) / United Nations system organization (e.g. UNESCO, UNICEF, FAO, etc.) / Donor organization (e.g. Foundations, private companies, etc.) / Other
If other, precise	
5. What type of work do you mainly do?	Research / Project and/or program evaluation / Project and/or program funding / Project and/or program development, coordination, management / Public policy development and/or public management / Advocacy (e.g., lobbying, community mobilization, etc.) / Media and communication (e.g., journalism, etc.) / Education and/or professional training / Other
If other, precise	
6. What level of decision making do you feel you have in your profession to influence or direct the strategic or policy choices of your organization?	Likert 5 points (Important)

7. How many years of experience do you have in the industry in which you work?	0 – 5 / 6 – 10 / 11 – 20 / 21 and more / I do not wish answer
Origin and migratory trajectory	
8. In which country were you born?	Belgium / Benin / Burkina Faso / Burundi / Cameroun / Canada / Comores / Ivory Coast / Djibouti / Gabon / Guinea / Equatoriale guinea / Haïti / France / Luxembourg / Madagascar / Mali / Monaco / Niger / Rwanda / central african republic / Republic of the Congo / Senegal / Seychelles / Switzerland / Tchad / Togo / Vanuatu / Other / I do not wish answer
If other, precise	
9. In which country do you live?	Belgium / Benin / Burkina Faso / Burundi / Cameroun / Canada / Comores / Ivory Coast / Djibouti / Gabon / Guinea / Equatoriale guinea / Haïti / France / Luxembourg / Madagascar / Mali / Monaco / Niger / Rwanda / central african republic / Republic of the Congo / Senegal / Seychelles / Switzerland / Tchad / Togo / Vanuatu / Other / I do not wish answer
If other, precise	
10. In which country did you obtain your highest degree?	Belgium / Benin / Burkina Faso / Burundi / Cameroun / Canada / Comores / Ivory Coast / Djibouti / Gabon / Guinea / Equatoriale guinea / Haïti / France / Luxembourg / Madagascar / Mali / Monaco / Niger / Rwanda / central african republic / Republic of the Congo / Senegal / Seychelles / Switzerland / Tchad / Togo / Vanuatu / Other / I do not wish answer
If other, precise	
**Knowledge and opinion on containment and infectious diseases (before reading)**	
About infectious disease outbreaks:	
11. How familiar are you with this topic?	Likert 5 points (Familiar)
12. How effective do you think containment measures are in containing them?	Likert 5 points (Effective)
13. In your opinion, how effective do your colleagues/workers feel the containment measures are in containing them?	
14. To the best of your knowledge, what is your opinion on the strength of the research evidence to support the use	Likert 5 points (Strong)

of containment measures to contain them?	
**Reading**	
15. How did you read the document?	I have read the whole document / I have read some parts / I have read almost nothing
Three scenarios to choose from to analyze the social norm*	
This document has been read by 458 people worldwide. Here is the opinion left by the readers: 325 (71%) people found it "excellent", 80 (17%) people found it "good", 21 (5%) people found it "average", 32 (7%) people found it "bad".<br>How would you rate this document?	Poor (=1) / Fair (=2) / Good (=3) / Excellent =4) / 0 = Don't know
This document has been read by 158 people in your country. Here is the opinion left by the readers: 112 (71%) people found it "excellent", 26 (17%) people found it "good", 8 (5%) people found it "average", 12 (7%) people found it "bad"<br>How would you rate this document?	
For the moment, this document is in test phase, nobody has given his opinion on its quality<br>How would you rate this document?	
**Knowledge and opinion on containment and infectious diseases (after reading)**	
16. After reading, how effective do you think containment measures are in containing infectious diseases?	Likert 5 points (Effective)
17. After reading, what is your opinion on the strength of the research evidence to support the use of containment measures to contain them?	Likert 5 points (Strong)
**Perception of the authoring organization**	
18. Can you identify the organization that wrote the document you read, please?	X
19. Do you know this organization?	Yes / No / I don't know
20. Are you used to receive information, newsletters or do you consult its news regularly?	Not at all / Rather not / Neutral / Rather / Totally
21. How much do you appreciate this organization?	
22. How trustworthy do you find her?	
23. How much do you think your colleagues / professional circle appreciate this organization?	
**Quality of knowledge**	

24. The content of the document is relevant to my work	Likert 5 points (Agree)
25. The content of the document is consistent with my professional values	
26. The content of the document seems to take into account a range of perspectives, not just that of the author	
27. The content of the document is easy to understand	
28. The level of detail provided in the document is appropriate	
29. The methodology presented in the document appears to be robust	
30. The argument presented in the document leading to the recommendations is convincing	
31. The visual presentation of the document is attractive	
32. The length of the document is adequate	
33. The proposed recommendations are clear	
**Use of the policy brief**	
34. Reread the document	
35. Send the document to someone else	
36. Share key messages of the document with colleagues/acquaintances	
37. Discuss this document with colleagues/acquaintances	
38. Write a blog post or article on the topic covered in the documen	
39. Read the complete studies on which the document is based if provided to me	
40. Find other information related to the topic of the document	
41. Cite this document in my reports or documents on the subject if I have the opportunity	
42. Change my opinion on the effectiveness of measures to control infectious disease outbreaks	
43. Change my current policies or practices with respect to the topic of containment measures to contain infectious disease outbreaks	

44. Développer ou commanditer de nouvelles études sur le sujet des mesures de confinement pour endiguer les épidémies de maladies infectieuses	
**Other**	
45. How confident are you in your ability to read, understand and interpret scientific studies?	
46. To what extent do the results of scientific studies generally influence or inform your practices?	

*This question was about testing social norms, we finally didn't use it but we verified that it didn't infer in our current analysis
