# Supplementary material for: When the messenger is more important than the message: an experimental study of evidence use in francophone Africa
Source: Health Res Policy Syst. 2022 May 26;20:57. doi: 10.1186/s12961-022-00854-x (PMC9134721; doi:10.1186/s12961-022-00854-x)
Supplement: Supplementary file 4 — Additional file 4: Description of the participants. [file 12961_2022_854_MOESM4_ESM.docx]

Additional file 4: Description of the participants

|  | N | % |
| --- | --- | --- |
| General characteristics |  |  |
| Gender |  |  |
| Male | 159 | 68.24 |
| Female | 72 | 30.90 |
| Other | 1 | 0.43 |
| Missing / Do not wish to answer | 1 | 0.43 |
| Age |  |  |
| 1- Less than 25 years old | 8 | 3.43 |
| 2- 26-35 years old | 67 | 28.76 |
| 3- 36-45 years old | 81 | 34.76 |
| 4- 46-55 years old | 48 | 20.60 |
| 5- 6-65 years old | 0 | 0.00 |
| 6- Over 65 years old | 27 | 11.59 |
| Missing / Do not wish to answer | 2 | 0.86 |
| Diplôme |  |  |
| 1- Under graduate | 5 | 2.15 |
| 2- Graduate 1st cycle (Bachelor) | 21 | 9.01 |
| 3- Graduate 2nd cycle (Master) | 95 | 40.77 |
| 4- Post graduate 3rd cycle (Doctorate) | 110 | 47.21 |
| Missing / Do not wish to answer | 2 | 0.86 |
| Profession characteristics |  |  |
| Organization in which the participant practices |  |  |
| 1- Academic institution or research center | 67 | 28.76 |
| 2- Government or ministry | 29 | 12.45 |
| 3- Government agencies (e.g. Institute of Public Health. etc.) | 17 | 7.30 |
| 4- Health center (e.g. hospitals. clinics. etc.) | 22 | 9.44 |
| 5- Non-governmental organization | 57 | 24.46 |
| 6- Civil society organization (local or international) | 11 | 4.72 |
| 7- United Nations system organization (e.g. UNESCO. UNICEF. FAO. etc.) | 11 | 4.72 |
| 8- Funding agency (e.g. foundations. private companies. etc.) | 6 | 2.58 |
| 9- Consulting firm | 6 | 2.58 |
| 10- Other | 7 | 3.00 |
| Sector of the profession |  |  |
| 1- Research | 57 | 24.46 |
| 2- Project and/or program evaluation | 12 | 5.15 |
| 3- Financing of projects and/or programs | 10 | 4.29 |
| 4- Development. coordination. management of projects and/or programs | 70 | 30.04 |
| 5- Public policy development and/or public management | 7 | 3.00 |
| 6- Advocacy (e.g. lobbying. community mobilization. etc.) | 4 | 1.72 |
| 7- Media and communication (e.g. journalism. etc.) | 6 | 2.58 |
| 8- Education and/or professional training | 13 | 5.58 |
| 9- Health professional | 43 | 18.45 |
| 10- Other | 11 | 4.72 |
| Sense of autonomy in the profession |  |  |
| Not at all | 18 | 7.73 |
| Somewhat not | 38 | 16.31 |
| Neutral | 14 | 6.01 |
| Somewhat | 98 | 42.06 |
| Totally | 60 | 25.75 |
| Missing / Do not wish to answer | 5 | 2.15 |
| Feeling of being able to influence or direct decisions within your organization |  |  |
| Not at all | 11 | 4.72 |
| Somewhat not | 32 | 13.73 |
| Neutral | 43 | 18.45 |
| Somewhat | 115 | 49.36 |
| Totally | 25 | 10.73 |
| Missing / Do not wish to answer | 7 | 3.00 |
| Experience in the profession |  |  |
| 0-5 years | 66 | 28.33 |
| 6-10 years of age | 52 | 22.32 |
| 11-20 years old | 70 | 30.04 |
| Over 21 years old | 43 | 18.45 |
| Missing / Do not wish to answer | 2 | 0.86 |
